# Remimazolam for general anesthesia in a patient with aortic stenosis and severe obesity undergoing transcatheter aortic valve implantation

**DOI:** 10.1186/s40981-024-00716-1

**Published:** 2024-05-27

**Authors:** Atsushi Kainuma, Ayaka Koyama, Mao Kinoshita, Jun Iida, Teiji Sawa

**Affiliations:** Department of Anesthesiology, Kyoto Prefectural University of Medicine, 465 Kajii-Cho, Kamigyoku, Kyoto 602–8566 Japan

**Keywords:** Remimazolam, Obesity, Transcatheter Aortic Valve Implantation, General Anesthesia, Aortic valve stenosis

## Abstract

**Introduction:**

There is currently limited research on the clinical use of remimazolam in severely obese patients. In this report, we describe the anesthesia management of transcatheter aortic valve implantation (TAVI) in a severely obese patient using remimazolam.

**Case description:**

A 76-year-old woman (height 1.54 m; total body weight 104 kg; body mass index 43.9 kg/m^2^) was scheduled for TAVI via the femoral artery approach for aortic valve stenosis. Preoperative echocardiography showed an aortic valve peak flow of 4.0 m/s and an effective orifice area of 0.75 cm^2^. Anesthesia induction was performed with a bolus dose of 100 μg fentanyl, 15 mg remimazolam, 60 mg rocuronium, and a continuous infusion of remifentanil at 0.4 mg/h. Intraoperatively, remimazolam was administered at a rate of 35 mg/h. General anesthesia management was completed without any complications, although the patient required temporary catecholamine and inhalation anesthesia assistance due to hemodynamic changes.

**Conclusion:**

Owing to its pharmacological advantages, remimazolam may be an option for anesthesia induction and maintenance in severely obese patients with unstable circulation.

## Background

Remimazolam has a rapid onset, less circulatory suppression, a short and predictable duration of sedation, and the ability to be antagonized using flumazenil [1–3]. These pharmacological features make remimazolam a potentially useful anesthetic for severely obese patients and those with unstable circulation [4]. Despite its potential benefits, there is currently limited research on the clinical use of remimazolam in severely obese patients. In this report, we describe the anesthetic management of transcatheter aortic valve implantation (TAVI) in a severely obese patient using remimazolam.

## Case presentation

A 76-year-old woman (height 1.54 m; total body weight (TBW) 104 kg; body mass index 43.9 kg/m^2^; ideal body weight (IBW) 47 kg; and adjusted body weight (ABW) [5] 69.7 kg) was scheduled for TAVI via the femoral artery approach for aortic valve stenosis. Preoperative laboratory investigations revealed an estimated glomerular filtration rate of 38 mL/min/1.73 m^2^, aspartate aminotransferase of 32 U/L, alanine aminotransferase of 11 U/L, and a total bilirubin level of 1.08 mg/dL. Pulmonary function tests showed a forced expiratory volume in one second at 72% of the predicted value and a vital capacity at 82% of the predicted value. Preoperative echocardiography showed a diastolic/systolic diameter of 52/34 mm, left ventricular ejection fraction of 64.6%, mild-to-moderate mitral regurgitation, mild aortic regurgitation, an aortic valve peak flow of 4.0 m/s, and an effective orifice area of 0.75 cm^2^.

**Fig. 1:**
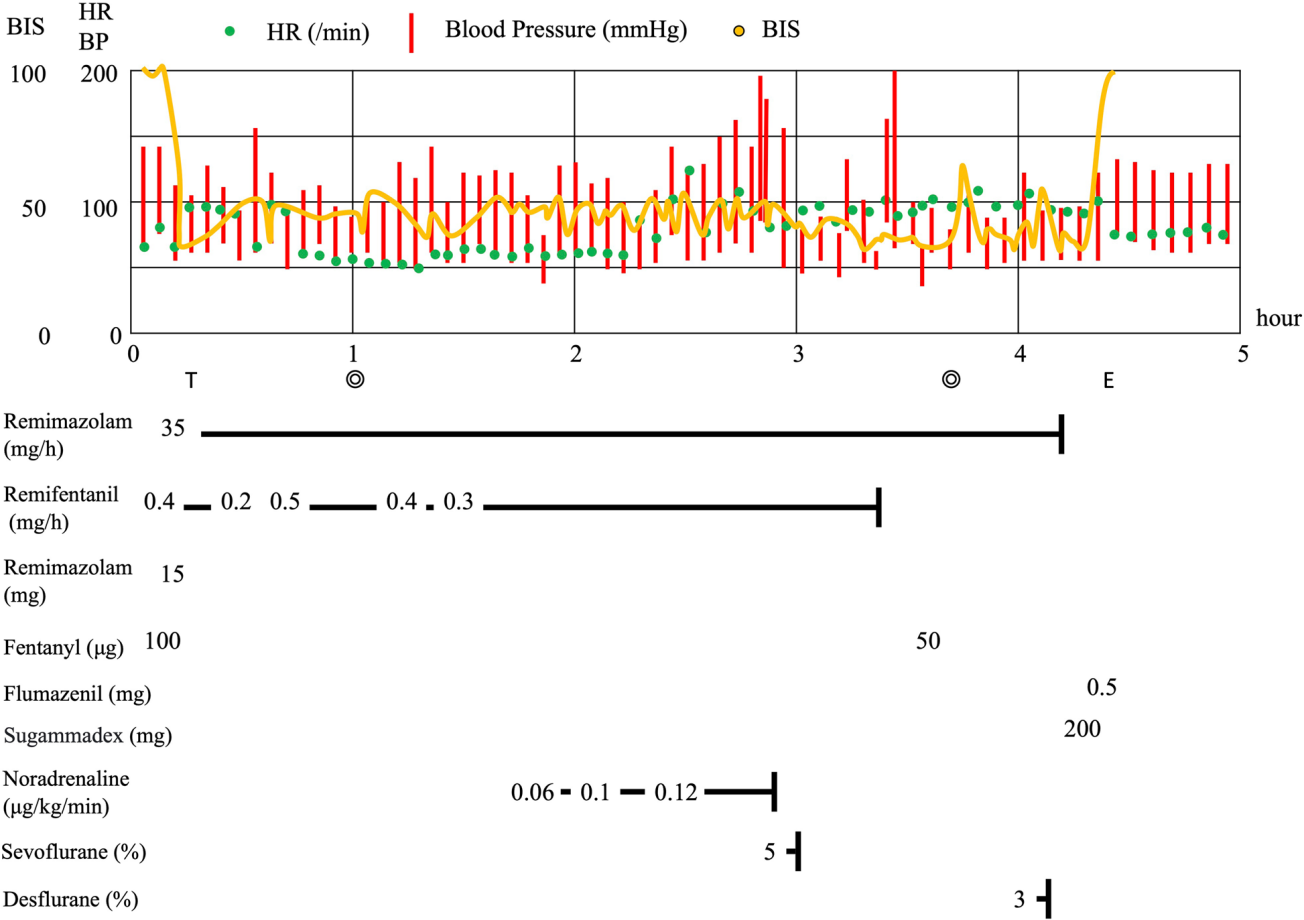
Anesthesia record. HR heart rate, ABP arterial blood pressure, BIS, The bispectral index, T tracheal intubation, E tracheal extubation

Hemodynamic record is shown in Fig. 1. Her oxygen saturation was 94% in room air in the operation theater. Anesthesia induction was performed with a bolus of 100 μg fentanyl and a continuous dose of 0.4 mg/h of remifentanil, followed 2 min later by a 15-mg bolus of remimazolam. The bispectral index (BIS) was in the 40 s immediately after remimazolam induction. Subsequently, she received 60 mg of rocuronium and was intubated using a McGrath MAC videolaryngoscope (Medtronic, Dublin, Ireland). Intraoperatively, remimazolam was administered at a dose of 35 mg/h. Additionally, noradrenaline 0.06 − 0.12 μg/kg/min was required for maintaining blood pressure until the aortic valve implantation, after which noradrenaline was discontinued and sevoflurane was administered to suppress an increase in BP.

The operative times were 1 h and 37 min. Following the completion of the surgery, desflurane was administered for 1 min as the BIS value temporarily increased to 70. Prior to extubation, remifentanil was discontinued, and the concentration of exhaled anesthetic gas was 0. The patient was administered 200 mg of sugammadex as the train-of-four count was 4. Thirty minutes after remifentanil was discontinued, and 5 min after remimazolam was discontinued, the patient exhibited a pharyngeal reflex and spontaneous breathing. Immediately thereafter, flumazenil 0.5 mg was administered, and the patient was successfully extubated. The patient’s conscious state after extubation was fully awake and well-oriented. The patient was discharged from the intensive care unit on postoperative day one without complications.

## Discussion

Administering remimazolam to severely obese patients based on TBW may result in excessively high doses, complicating its use in clinical practice. In our case, the maintenance dose was calculated based on the ABW [5]. The ABW was calculated using the following formula: The patient's IBW was 46.8 kg and ABW was 69.7 kg.

$$\text{IBW}=45.4+0.89\times\left(\text{Height}-152.4\right)+4.5\times\left(1-\text{Gender}\right)$$  

$$\text{ABW}=\text{IBW}+0.4\times \left(\text{TBW}-\text{IBW}\right)$$

IBW, ideal body weight; ABW, adjusted body weight (kg); TBW, total body weight (kg); TBW, total body weight (kg); Height (cm); Gender, 0 for males, 1 for females.

Continuous infusion of remimazolam 6 mg/kg/h was used for induction of general anesthesia in a morbidly obese patient [4]. In a previous study involving patients aged 60–80 years, the induction dose ranged from 0.14 to 0.19 mg/kg [6]. Based on that report, the attending anesthesiologist administered remimazolam for induction at approximately 0.2 mg/kg of ABW. In our case, the BIS rapidly decreased with a bolus dose of 15 mg during anesthesia induction. To maintain general anesthesia, the patient received remimazolam at 0.5 mg/kg/h of ABW. In a previous report, remimazolam was maintained at 0.56 mg/kg/h of TBW for general anesthesia in ASA-PS3 patients [5, 7]. Notably, female patients may require a higher remimazolam infusion rate than male patients. Patients with a higher ASA-PS classification generally need a lower infusion rate than those with lower classifications. Additionally, patients with increased weight exhibit higher drug concentrations than those with lower weight when administered the same dose per kg [5]. Compared with previous reports, we used a lower maintenance dose of remimazolam. Another report indicated that the maintenance dose of remimazolam ranged from 0.3 to 0.5 mg/kg/h when used with remifentanil [8]. In severely obese patients, there is a risk of overdosing remimazolam based on TBW. Thus, at the discretion of the attending anesthesiologist, the maintenance dose of remimazolam in this case was set at 0.5 mg/kg/h based on ABW.

In our case, increased blood pressure after aortic valve implantation necessitated prompt blood pressure reduction using sevoflurane. Given that remimazolam has a lesser impact on circulatory suppression, it is imperative to contemplate strategies for managing elevated blood pressure, especially in instances of substantial improvement in circulation or during procedures involving severe surgical invasiveness.

In our case, flumazenil was used to achieve effective antagonism after the pharyngeal reflex, and spontaneous breathing was observed. Using a high dose of remimazolam and antagonizing it with a high dose of flumazenil may result in inadequate antagonism. Another risk associated with using high doses of flumazenil is the potential for seizures following flumazenil administration [9, 10]. However, the appropriate dose of flumazenil used as an antagonist remains unclear. In addition, the metabolism of remimazolam under conditions of low cardiac output or in the presence of valve disease remains uncertain. Accumulation should also be considered with long-term (> 24 h) use of remimazolam (ONO-2745–04) [11].

We determined the depth of anesthesia based on BIS values in this case. Previous research has indicated that BIS values during administration of remimazolam at appropriate doses are generally higher than those observed with other anesthetics, with some patients exhibiting BIS values greater than 60 [12, 13]. Due to individual differences in remimazolam use, it is necessary to quantitatively assess sedation, including electroencephalogram (EEG) monitoring.

In future cases with severely obese patients, remimazolam dosing based on ABW could help prevent excessive dosing. EEG monitoring, such as BIS, is a useful tool for assessing sedation levels during general anesthesia with remimazolam. Appropriate pain management for surgery is essential. We recommend the use of antagonist agents for remimazolam; however, the optimal timing and dosage remain undefined. In this case, the administration of the antagonist was initiated after signs of awakening were observed. Additionally, strategies for managing hemodynamic changes are necessary; these should include the use of inhaled anesthesia or calcium channel blockers to manage sudden blood pressure increases.

We managed anesthesia with remimazolam for TAVI in a severely obese patient with a BMI > 40. Owing to its pharmacological advantages, remimazolam may be an option for anesthesia induction and maintenance in severely obese patients with unstable circulation.

## Data Availability

All data are included in this article.
